# Heterogeneity-Aware Personalized Federated Neural Architecture Search

**DOI:** 10.3390/e27070759

**Published:** 2025-07-16

**Authors:** An Yang, Ying Liu

**Affiliations:** College of Information Science and Electronic Engineering, Zhejiang University, Hangzhou 310027, China; 12031073@zju.edu.cn

**Keywords:** neural architecture search, neural network, federated learning, personalization

## Abstract

Federated learning (FL), which enables collaborative learning across distributed nodes, confronts a significant heterogeneity challenge, primarily including resource heterogeneity induced by different hardware platforms, and statistical heterogeneity originating from non-IID private data distributions among clients. Neural architecture search (NAS), particularly one-shot NAS, holds great promise for automatically designing optimal personalized models tailored to such heterogeneous scenarios. However, the coexistence of both resource and statistical heterogeneity destabilizes the training of the one-shot supernet, impairs the evaluation of candidate architectures, and ultimately hinders the discovery of optimal personalized models. To address this problem, we propose a heterogeneity-aware personalized federated NAS (HAPFNAS) method. First, we leverage lightweight knowledge models to distill knowledge from clients to server-side supernet, thereby effectively mitigating the effects of heterogeneity and enhancing the training stability. Then, we build random-forest-based personalized performance predictors to enable the efficient evaluation of candidate architectures across clients. Furthermore, we develop a model-heterogeneous FL algorithm called heteroFedAvg to facilitate collaborative model training for the discovered personalized models. Comprehensive experiments on CIFAR-10/100 and Tiny-ImageNet classification datasets demonstrate the effectiveness of our HAPFNAS, compared to state-of-the-art federated NAS methods.

## 1. Introduction

In the digital era, data stands as a critical linchpin. With the advancement of the Internet of Things (IoTs), the proliferation of edge devices—such as mobile phones and wearable devices—has led to an exponential increase in private data generated from distributed sources. Considering data privacy, traditional centralized learning fails to effectively leverage distributed multi-party data, commonly manifested as *data islands*. Federated learning (FL), which enables multiple participants (clients) to collectively train a shared model while preserving data locality, has emerged [1].

In real-world applications, statistical heterogeneity and resource heterogeneity are two key practical challenges for FL. The former arises from the fact that clients typically hold diverse and non-IID data. The latter reflects the varying computational and memory resources across clients. However, traditional FL typically assumes a shared model architecture for all clients and neglects clients’ personalized requirement. Therefore, it is promising to develop personalized models that comply with local resource constraints and suit local data distributions for clients in heterogeneous FL systems [2].

Neural architecture search (NAS) offers a promising solution for automatically designing model architectures by exploring a predefined model architecture search space using a specific search strategy [3,4,5,6,7,8,9]. However, conventional NAS methodologies incur prohibitive computational overhead, necessitating the complete training of every candidate model from scratch to facilitate performance-based search process. To alleviate this computational burden, the weight-sharing paradigm has been introduced, giving rise to one-shot NAS methods [3,4,6,7,8,9]. Among these, sample-based one-shot NAS is particularly well-suited for designing personalized models for multiple heterogeneous clients in FL [6,7,8,9]. Specifically, it involves training a weight-sharing supernet that encompasses all candidate models (subnets). Once the supernet is fully trained, candidate architectures can directly inherit their weights for efficient performance evaluation, eliminating the need for separate, stand-alone training. Typically, the supernet is trained by sampling subnets and updating their associated shared weights [6,7]. In FL, this training process can be naturally performed in a resource-aware manner [10,11,12,13]. Specifically, sampled subnets are assigned to compatible clients based on resource constraints, thereby leveraging the distributed computational resources of FL systems. Nevertheless, each subnet is inherently coupled with the local data distributions of its compatible clients. In other words, subnets are trained on non-IID data. This exacerbates the existing gradient conflicts when subnets update their shared weights within the supernet, thereby destabilizing the supernet training. As a result, the performance ranking of subnets with inherited weights becomes unstable and inaccurate, which further degrades the overall effectiveness of federated NAS [14,15].

To address this challenge, we propose a resource-resilient federated supernet training strategy to enhance the supernet’s training stability. Furthermore, we propose a heterogeneity-aware personalized federated neural architecture search method (HAPFNAS). Our contributions are summarized as follows:

- We propose a resource-resilient federated supernet training strategy based on ensemble distillation in heterogeneous FL systems. Specifically, we use lightweight knowledge models to distill knowledge from FL clients to the server-side supernet, thereby alleviating the exacerbated gradient conflicts arising from the coupling of resource heterogeneity and statistical heterogeneity.
- We propose a predictor-guided personalized evolutionary search algorithm. Specifically, we establish a performance predictor using random forest to quickly predict the performance of candidate architectures on a target client, thus eliminating time-consuming inference evaluation and accelerating the search process.
- Additionally, we develop a model-heterogeneous FL algorithm, termed heteroFedAvg, to facilitate collaborative learning among searched personalized models that originate in the same search space.

The rest of this paper is organized as follows. In Section 2, we provide an introduction to some related works. In Section 3, we present some preliminaries of one-shot NAS and clarify the motivation of this study. In Section 4, we provide a comprehensive elaboration on the proposed HAPFNAS. In Section 5, we conduct extensive experiments to verify the effectiveness of HAPFNAS. Finally, some conclusions are drawn in Section 6.

## 2. Related Works

### 2.1. Federated Neural Architecture Search

The primary objective of NAS is to establish an automated framework for designing neural network architectures that are both efficient and effective [3,5,6,7,8,9]. Federated NAS is dedicated to designing a shared model architecture or multiple personalized model architectures for multiple clients with or without a centralized server [10,11,12,13,16,17,18,19,20,21,22,23,24,25]. Existing works are mainly classified into two branches: gradient-based federated NAS [10,16,17,18,19,20,21,22,23,24] and sample-based one-shot federated NAS [11,12,13,25].

The gradient-based federated NAS embeds weight coefficients (architecture parameters) that signify the importance of candidate operations into the weight-sharing supernet, allowing the architecture parameters to be updated using traditional gradient descent along with the federated learning process and thereby enabling an exploration of the search space. The inherent extensibility of gradient-based optimization to federated learning frameworks has motivated numerous derivative research efforts in this domain. FedNAS [16], DFNAS [17], and FDNAS [10] extend centralized gradient-based MiLeNAS [5], DSNAS [4], and ProxylessNAS [3] to federated scenarios, respectively. DPNAS [18] and DP-FNAS [19] improve privacy protection by adding noise and provide theoretical privacy guarantees. FedPNAS [20], Spider [21], and FDNAS+ [22] focus on providing personalized model architectures to emphasize the differences in tasks on FL clients. HANF [23] incorporates hyperparameter optimization into federated NAS. FedAutoMRI [24] broadens the application of gradient-based federated NAS to encompass MRI tasks. Although the gradient-based federated NAS methods demonstrate high inclusiveness and flexibility towards privacy protection and personalization in FL, they require the transmission of a giant over-parameterized supernet, which is impractical in bandwidth-limited IoT systems.

On the other hand, sample-based one-shot federated NAS concentrates on fully training a one-shot supernet as a proxy performance evaluator to simultaneously maintain numerous candidate architectures, enabling efficient search across multiple scenarios, e.g., personalized search for heterogeneous clients in FL. Generally, the one-shot supernet is trained through sampling subnets and updating the associated shared weights. In federated scenarios, the updates for different subnets can be naturally distributed to FL clients. RT-FedEvoNAS [25] orchestrates both supernet training and evolutionary search simultaneously by randomly assigning population individuals (subnets) to random clients for training to update the shared weights within the supernet. However, it fails to take resource heterogeneity into consideration. DCFMS [11], FedorAS [12], and DC-NAS [13] employ similar resource-aware federated supernet training strategies, adaptively assigning sampled subnets to suitable clients with matching resources. Even though this resource-aware strategy leverages distributed computational resources, it neglects the impact of the coupling between resource heterogeneity and statistical heterogeneity on the supernet training. As a result, it tends to exacerbate the instability of supernet training and further diminishes accuracy in ranking the performance of candidate subnets, which is important to NAS.

In contrast to the aforementioned methods, in this work, we propose a resource-resilient federated supernet training strategy to alleviate the gradient conflicts caused by the coupling between resource heterogeneity and statistical heterogeneity, targeted for enhancing the supernet’s training stability in heterogeneous FL systems.

### 2.2. Model-Heterogeneous Federated Learning

Besides statistical heterogeneity, the proliferation of edge devices is escalating the challenges posed by resource heterogeneity. Consequently, model-heterogeneous FL has emerged as a rapidly growing research focus in the machine learning community [26,27,28,29,30,31,32,33,34,35,36]. The primary challenge arising from heterogeneous models is that the traditional model aggregation using FedAvg [1] is no longer applicable. Thus, figuring out how to achieve information fusion across clients and information exchange between clients and server has become the most critical problem to address.

The current prevalent solution uses knowledge distillation to achieve model-agnostic knowledge transfer. FedMD [26], FedCD [27], KT-pFL [28], Cronus [29], Def-KT [30], and FD+FAug [31] compel predicted logits of client models to approximate the fusion of predicted logits from the other or all clients, thereby achieving information exchange across clients. FedBD [32] employs ensemble distillation to amalgamate the knowledge of heterogeneous models on clients into the server-side model. The fused knowledge is then distilled back to client models from the server-side model, thus establishing a bidirectional distillation channel between server and clients. FedDF [33] and FedZKT [34] directly implement mutual distillation between the server-side model and heterogeneous client models on the server. Diverging from the aforementioned methods, FedKEMF [35] and RaFL [36] construct knowledge models for the extraction of knowledge from heterogeneous client models. Subsequently, these knowledge models act as knowledge carriers to facilitate the traditional average aggregation for information fusion and exchange.

In contrast to the aforementioned methods, for the personalized client models discovered via NAS, we exploit the homogeneity of these models that originate in the same search space and then propose a model-heterogeneous aggregation algorithm to facilitate collaborative learning.

## 3. Preliminaries and Motivation

In one-shot NAS [3,6,8,9], the search space A is usually formulated as a weight-sharing supernet with shared weights *W*. In this way, each candidate architecture α∈A corresponds to a subnet, whose weights are a subset of the supernet weights, denoted by $W_{\alpha }$. The canonical one-shot NAS is formulated as a bi-level optimization problem:

(1) $$\begin{array}{cc} \alpha^{*} & =\arg\min_{\alpha \in A}L\left(W_{\alpha }^{*};D^{val}\right)\\ s.t.\;W_{\alpha }^{*} & =\arg\min_{W_{\alpha }}L\left(W_{\alpha };D^{trn}\right), \end{array}$$

where $D^{trn}$ and $D^{val}$ denote the training dataset and the validation dataset, respectively, and $L\left(\cdot \right)$ denotes the loss function, e.g., the cross-entropy loss for image classification. It is non-trivial to solve the optimization problem (1) with coupled *W* and α. Instead, sample-based one-shot NAS decouples this problem by first training the supernet and then conducting architecture search [6,8,9]. Specifically, the one-shot supernet is trained by iteratively sampling subnets and updating their associated shared weights, based on a predetermined sampling rule α∼Γ(A) [6,8,9]. This yields the following optimization objective for the supernet:

(2) $$\min_{W}\sum_{\alpha ∼\Gamma (A)}\left[L\left(W_{\alpha };D^{trn}\right)\right].$$

**Figure 1:**
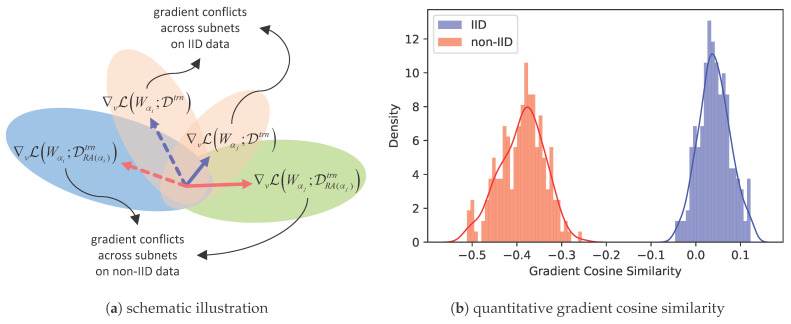
Gradient conflicts in shared weights when subnets are trained on IID vs. non-IID data.

Once the supernet is fully trained, the performance of subnets inheriting shared weights is treated as a proxy for their ground truth performance, thereby significantly accelerating the search process. However, the weight-sharing paradigm inherently induces deep coupling between weights and architectures. This causes gradient conflicts when updating the shared weights $v=W_{\alpha_{i}}⋂W_{\alpha_{j}}$ between any two subnets $\alpha_{i}$, $\alpha_{j}$, as indicated by two blue arrows in Figure 1a. Furthermore, it destabilizes supernet training and induces unstable and inaccurate performance ranking of candidate architectures, thereby posing a grave threat to NAS [14,15].

**Figure 2:**
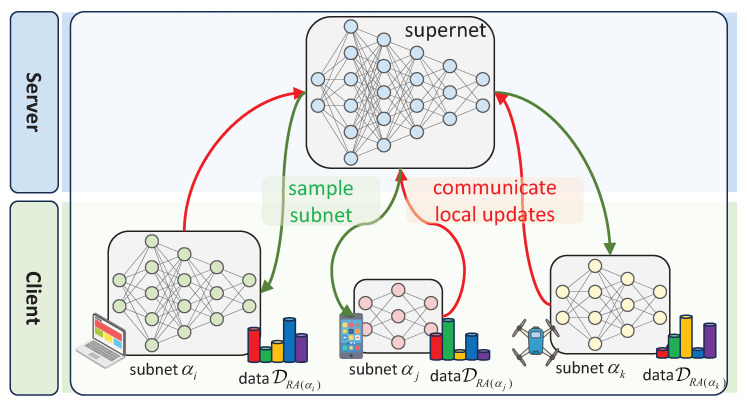
Resource-aware federated supernet training strategy in heterogeneous FL systems.

When sample-based one-shot NAS is transferred to FL scenarios—comprising one server node and *N* clients with non-IID data ${\left\{D_{i}\right\}}_{i=1:N}$—most federated NAS methods have naturally adapted (2) to a resource-aware federated supernet training strategy, as depicted in Figure 2. The core of this strategy is to assign sampled subnets ${\left\{\alpha_{i}\right\}}_{i=1:k}$ to suitable and compatible clients based on computational resources [11,12,13], thereby making full use of distributed resources. Assuming that a subnet α is assigned to client RA(α)∈{1,2,…,N} in the resource-aware assignment strategy, the resource-aware federated supernet training strategy can be formulated as follows:

(3) $$\min_{W}\sum_{\alpha ∼\Gamma (A)}\left[L\left(W_{\alpha };D_{RA(\alpha )}^{trn}\right)\right].$$

However, this resource-aware strategy overlooks the impact of the coexistence of resource heterogeneity and statistical heterogeneity on supernet training. Compared to the centralized setting in  (2), this resource-aware strategy induces a deep coupling between a sampled subnet α and the data distribution $D_{RA(\alpha )}$ of client RA(α). In other words, sampled subnets are trained on non-IID data. As a result, this coupling exacerbates gradient conflicts between any two subnets $\alpha_{i}$, $\alpha_{j}$, with $RA\left(\alpha_{i}\right)\neq RA\left(\alpha_{j}\right)$ updating shared weights $v=W_{\alpha_{i}}⋂W_{\alpha_{j}}$, as indicated by two red arrows in Figure 1a.

To empirically validate this point, we randomly sample two subnets with shared weights and compute the cosine similarity of their gradients to quantify gradient conflicts when trained on IID and non-IID data distributions, respectively. As shown in Figure 1b, the results reveal that subnets exhibit significantly lower gradient cosine similarity on shared weights under non-IID data distribution than that under IID settings, which indicates more severe gradient conflicts caused by statistical heterogeneity. In summary, resource-aware federated supernet training strategy exacerbates the gradient conflicts that inherently exist in supernet, degrades the stability of supernet training, and impairs the reliability of performance ranking among subnets.

## 4. Methods

**Figure 3:**
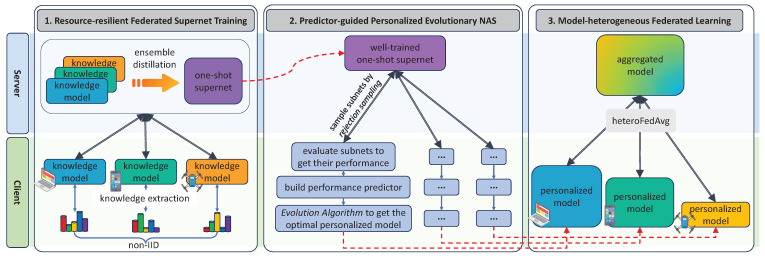
The framework of the proposed heterogeneity-aware personalized federated neural architecture search.

In this section, the detailed design of HAPFNAS is presented. As shown in Figure 3, our HAPFNAS consists of three pivotal parts: (1) *a resource-resilient federated supernet training strategy* for training a one-shot supernet; (2) *a predictor-guided personalized evolutionary search algorithm* targeted at searching for promising personalized models for FL clients based on a well-trained supernet; and (3) *a model-heterogeneous FL algorithm termed heteroFedAvg* to facilitate collaborative learning among clients’ searched personalized models. Each of these parts will be discussed sequentially in the following.

### 4.1. Resource-Resilient Federated Supernet Training

**Figure 4:**
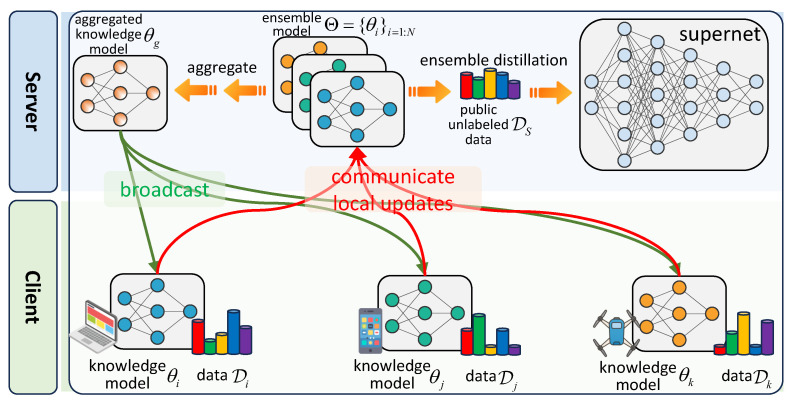
The proposed resource-resilient federated supernet training strategy in heterogeneous FL systems.

To avoid the negative impact of the coexistence of resource heterogeneity and statistical heterogeneity on supernet training, we aspire to preserve the centralized supernet optimization (2) while leveraging the private data distributed across FL clients. Towards this goal, we propose a resource-resilient federated supernet training strategy, which is schematically depicted in Figure 4. The detailed design is shown in the following.

#### 4.1.1. Knowledge Collection for Supernet Training

Firstly, considering the resource heterogeneity, we design a lightweight knowledge model $\theta_{g}$, which is a shared model between the server and clients in the FL. It is first broadcast to heterogeneous clients for extracting local knowledge based on their private data $D_{i}^{trn}$. The local training of $\theta_{i}$ on client *i* is formulated as follows:

(4) $$\min_{\theta_{i}}L\left(\theta_{i};D_{i}^{trn}\right).$$

After the local training, the clients transmit the updated knowledge models ${\left\{\theta_{i}\right\}}_{i=1:N}$ to the server. Then, the server aggregates all of the received knowledge models to update the global model as $\theta_{g}=\frac{1}{N}\sum_{i=1}^{N}\theta_{i}$, and then broadcast to clients in the next communication round.

#### 4.1.2. Supernet Training on the Server

Leveraging the ensemble of client-updated knowledge models $\Theta ={\left\{\theta_{i}\right\}}_{i=1:N}$, the supernet training can be carried out directly on the server based on ensemble distillation. Specifically, we distill the knowledge from the ensemble model Θ to the supernet via the public unlabeled dataset $D_{S}$ on the server. Note that here we focus on unlabeled data since it is difficult and/or expensive to collect a large amount of labeled data in many real-world applications. For any unlabeled sample $x\in D_{S}$, we compute the ensemble’s output as the average of client logits, i.e., $\Theta \left(x\right)=\frac{1}{N}\sum_{i=1}^{N}\theta_{i}\left(x\right)$. Then, in supernet training, all sampled subnets are trained to align with the above ensemble prediction. The corresponding supernet optimization problem is formulated as

(5) $$\min_{W}\sum_{\alpha_{i}∼\Gamma \left(A\right)}^{k}\left[L_{div}\left(W_{\alpha_{i}}∥\Theta ;D_{S}\right)\right],$$

where $L_{div}$ denotes the distillation loss function, e.g., Kullback–Leibler (KL) divergence [37].

Optimizing (5) can bypass the scheme that assigns subnets to heterogeneous clients used in the existing resource-aware strategy (see Figure 2). As a result, it effectively alleviates the gradient conflicts caused by the coupling between resource heterogeneity and statistical heterogeneity, thereby enhancing the stability of supernet training.

Furthermore, we incorporate the *sandwich sampling* rule and *inplace distillation* technique into our supernet training [6,7,38]. Consequently, the optimization problem (5) is reformulated as

(6) $$\min_{W}L_{div}\left(W_{\alpha_{l}}∥\Theta ;D_{S}\right)+L_{div}\left(W_{\alpha_{s}}∥W_{\alpha_{l}};D_{S}\right)+\sum_{\alpha_{i}∼U\left(A\right)}^{k}L_{div}\left(W_{\alpha_{i}}∥W_{\alpha_{l}};D_{S}\right).$$

Specifically, following the sandwich sampling rule, we sample the largest subnet $\alpha_{l}$, the smallest subnet $\alpha_{s}$, and *k* randomly sampled subnets ${\left\{\alpha_{i}\right\}}_{i=1:k}$ at each iteration. According to the inplace distillation, the aforementioned ensemble distillation is implemented solely for the largest subnet $\alpha_{l}$, as expressed by the first term of (6), whereas the smallest subnet $\alpha_{s}$ and other sampled subnets ${\left\{\alpha_{i}\right\}}_{i=1:k}$ are supervised by the largest subnet $\alpha_{l}$, represented by the second and third terms, respectively.

For clarity, the entire supernet training process is shown in Algorithm 1. The effectiveness of the proposed resource-resilient strategy will be discussed in Section 5.2.
**Algorithm 1** Resource-resilient Federated Supernet Training**Input**: Initialize the knowledge model $\theta_{g}$, supernet *W*, number of clients *N*, total round *T* **ClientUpdate**: 1:**receive** $\theta_{g}$ from **server** 2:$\theta_{i}$← solve optimization problem (4) 3:**communicate** $\theta_{i}$ with **server****ServerUpdate**: 1:**for** round t=1,2,…,T **do** 2:      **broadcast** $\theta_{g}$ to **clients** 3:      **for** each client *i* in parallel **do** 4:           $\theta_{i}$←**ClientUpdate**($\theta_{g}$) 5:      **end for** 6:      $\theta_{g}=\frac{1}{N}\sum_{i=1}^{N}\theta_{i}$ 7:      // train supernet 8:      $\Theta \leftarrow {\left\{\theta_{i}\right\}}_{i=1:N}$ 9:      $\alpha_{l}$, $\alpha_{s}$, ${\left\{\alpha_{i}\right\}}_{i=1:k}$←**Sandwich Sampling**(A)10:      *W*← solve optimization problem (6)11:**end for**

### 4.2. Predictor-Guided Personalized Evolutionary NAS

In sample-based one-shot NAS, the fully trained supernet serves as a proxy evaluator for candidate architectures. Specifically, the performance of subnets inheriting shared weights is treated as proxy for their ground truth performance, thus significantly accelerating the subsequent search process. However, candidate architectures with inherited weights cannot be directly used for evaluation. The fundamental limitation arises from the shared Batch Normalization (BN) statistics in supernet architectures, which fail to capture subnet-specific distribution characteristics [39]. To mitigate this discrepancy, it is common to recalibrate BN statistics for each candidate architecture before performance evaluation [40]. In our HAPFNAS, aiming at discovering personalized model architecture for each client, we recalibrate the BN statistics using the private data $D_{i}^{trn}$ of the target client *i*, rather than relying on the server-side $D^{S}$. This ensures that the recalibrated subnet aligns with the client’s local data distribution, thereby enabling accurate performance estimation on the target client. According to general experience, for one candidate architecture, calibrating BN statistics and then evaluating it typically takes just a few seconds. However, the search strategy typically explores thousands of candidates, thus posing an unaffordable computational burden and time consumption, notably for edge clients.

To address this, we introduce a performance predictor based on random forest to further accelerate the performance evaluation on each client [6]. Firstly, for the target client *i*, we sample a modest batch of candidate architectures that satisfy local resource constraints via *reject sampling*. These sampled architectures are then recalibrated on the training dataset $D_{i}^{trn}$ and evaluated on the validation dataset $D_{i}^{val}$. In this way, we obtain many architecture-performance pairs, which are used to train the random-forest-based performance predictor. Note that this trained predictor is personalized and tailored to the target client *i*. In this way, assisted by client-specific performance predictors, the performance of candidate architectures can be efficiently estimated with negligible computational cost, thereby enabling an efficient search process to discover promising personalized model architectures on each client.

In our HAPFNAS, we employ the evolutionary algorithm (EA) as the search strategy. Only the subnets within the initial population of the evolutionary process are used to train performance predictors. For clarity, the predictor-guided personalized evolutionary NAS algorithm is summarized in Algorithm 2.
**Algorithm 2** Predictor-guided personalized evolutionary neural architecture search on client *i***Input**: The well-trained trained supernet *W*, search space A, total generations $N_{gen}$, population size $N_{pop}$, mutation size $N_{m}$, crossover size $N_{c}$  1:Initialize population P by **Reject Sampling**(A) 2:**for** each candidate architecture α∈P **do** 3:      inherit shared weights $W_{\alpha }$ from **server**-side supernet 4:      recalibrate BN statistics of $W_{\alpha }$ on $D_{i}^{trn}$ 5:      loss(α)← evaluate α on $D_{i}^{val}$ 6:**end for** 7:// train random-forest-based performance predictor 8:*D*←${\left\{\alpha_{j},loss\left(\alpha_{j}\right)\right\}}_{j=1:N_{pop}}$ 9:**RFpredictor**← train predictor on *D*10:**for** generation $g=1,2,\ldots ,N_{gen}$ **do**11:      // get $N_{m}$ new compatible architectures that comply with local resource constraints by mutation12:      $P_{m}\leftarrow$**Mutation**(P)13:      // get $N_{c}$ new compatible architectures that comply with local resource constraints by crossover14:      $P_{c}\leftarrow$**Crossover**(P)15:      **for** each candidate architecture $\alpha \in P_{m}\cup P_{c}$ **do**16:           loss(α) ←**RFpredictor**(α) // get fitness17:      *P*← the best $N_{pop}$ architectures from $P\cup P_{m}\cup P_{c}$18:**return** the best personalized architecture $\alpha_{i}^{*}$

### 4.3. Model-Heterogeneous Federated Learning

Through the aforementioned heterogeneity-aware personalized architecture search, each client obtains their own personalized model architecture, denoted as ${\left\{\alpha_{i}^{*}\right\}}_{i=1:N}$. However, collaborative training across these heterogeneous models remains challenging, primarily because traditional model aggregation methods (e.g., FedAvg) are no longer readily applicable. Nonetheless, since all personalized models are discovered from the same search space, there exists an inherent structural alignment that enables the possibility of model aggregation. Notably, it is evident that all searched models include, at a minimum, the smallest subnet defined within the search space. This observation implies that each personalized model architecture can be conceptually decomposed into a shared or common component and a private one. For instance, given two model architectures $\alpha_{i}^{*}$ and $\alpha_{j}^{*}$, they can be decomposed into a shared subarchitecture $\alpha_{s}$ and private components $\alpha_{i}^{*}-\alpha_{s}$ and $\alpha_{j}^{*}-\alpha_{s}$, where the subtraction operator ‘−’ denotes structural pruning. Based on this decomposition, aggregating only the shared components while preserving the private ones is feasible. Furthermore, this aggregation can be naturally extended to aggregate the largest shared subarchitecture, denoted as $\alpha_{i}^{*}\cap \alpha_{j}^{*}$, rather than limiting aggregation to the smallest subnet $\alpha_{s}$.

**Figure 5:**
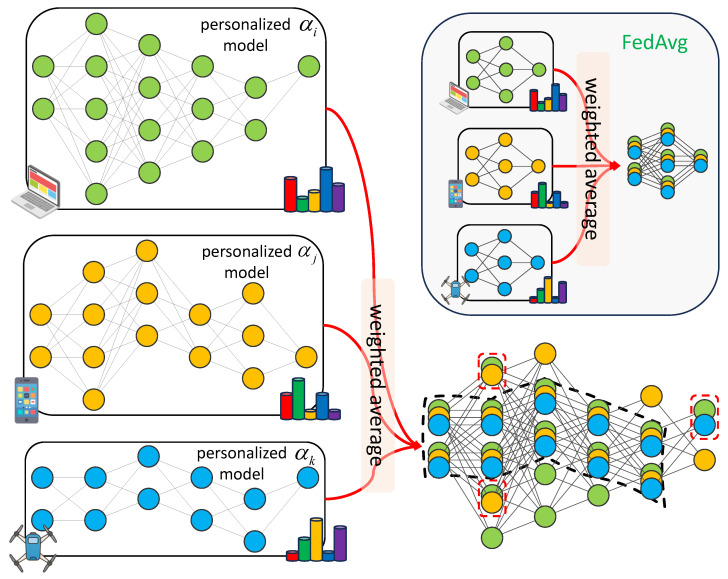
Model-heterogeneous aggregation across personalized client models in the proposed heteroFedAvg: aggregating the shared subarchitecture available among personalized models while preserving client-specific private components.

Building on this insight, and inspired by the weight-sharing paradigm in NAS, we propose a model-heterogeneous FL algorithm, termed heteroFedAvg, which is schematically illustrated in Figure 5. In the proposed heteroFedAvg, the aggregation among heterogeneous models actually depends on the way they share weights within the supernet. Specifically, similar to FedAvg, our heteroFedAvg directly aggregates the largest shared subarchitecture among all personalized models, as marked by the black dashed box in Figure 5. Moreover, for components shared by a subset of heterogeneous models, as indicated by the red dashed box in Figure 5, our heteroFedAvg also applies the weighted average aggregation to further enhance knowledge fusion across clients.

**Figure 6:**
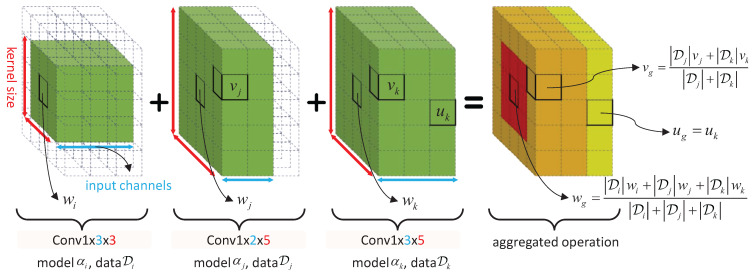
Aggregation across three heterogeneous convolution operations with varying kernel sizes and channel configurations from three heterogeneous client models. ‘Conv*O*x*I*x*K*’ denotes the convolution with *I* input channels, *O* output channels, and a kernel size of K×K. Colored cubes represent actual weight parameters, while transparent cubes are auxiliary visualizations to illustrate weight sharing within the supernet.

To clearly illustrate this aggregation process, we present an example of fine-grained parameter aggregation that involves three convolution operations from three clients’ personalized models, as shown in Figure 6. These convolutions differ in input/output channels and kernel sizes, to be specific ‘Conv1x3x3’, ‘Conv1x2x5’, and ‘Conv1x3x5’ from left to right in Figure 6. For each weight parameter *v* (indicated by the green cube in Figure 6), the weighted average aggregation based on local data sizes can be formulated as

(7) $$v_{g}=\sum_{i\in S_{v}}\frac{\left|D_{i}\right|}{\sum_{j\in S_{v}}\left|D_{j}\right|}v_{i},$$

where $S_{v}$ denotes the set of clients whose personalized model architectures contain parameter *v*.

## 5. Experiments

In this section, we first give the experimental setting. Then, we validate the effectiveness of the three main parts of our HAPFNAS method shown in Figure 3, respectively. After that, we perform HAPFNAS on CIFAR-10/100 [41] and Tiny-ImageNet [42] datasets in heterogeneous FL scenarios with different numbers of clients. Then, we evaluate the performance of the searched personalized model architectures to verify the effectiveness of HAPFNAS.

### 5.1. Experimental Setup

**Environment settings**:

All federated experiments are conducted on a single GeForce RTX 4090 GPU (NVIDIA Corporation, Santa Clara, CA, USA) using FederatedScope v0.3.0 [43] as the simulated federated learning platform. All neural network implementations are based on PyTorch v2.0.1, running on Ubuntu 22.04 LTS with CUDA Toolkit v12.1.

**Search space:**

We closely follow the MobileNetV3 [44]-like search space used in AttentiveNAS [6], which mainly consists of several searchable mobile inverted residual bottleneck blocks with variable kernel sizes, expansion ratios, and channel configurations. The input resolution is set to 32×32.

**Datasets:**

Our following experiments are based on three datasets, CIFAR-10, CIFAR-100, and Tiny-ImageNet. CIFAR-10/100 consists of 50,000 training images and 10,000 test images, evenly distributed across 10/100 categories. Compared to CIFAR-10/100, Tiny-ImageNet is more challenging, consisting of 200 classes with 500 training, 50 validation, and 50 test images per class. Since Tiny-ImageNet does not publicly disclose labels for the test images, the original validation dataset is considered as the test dataset.

**Federated settings:**

**Figure 7:**
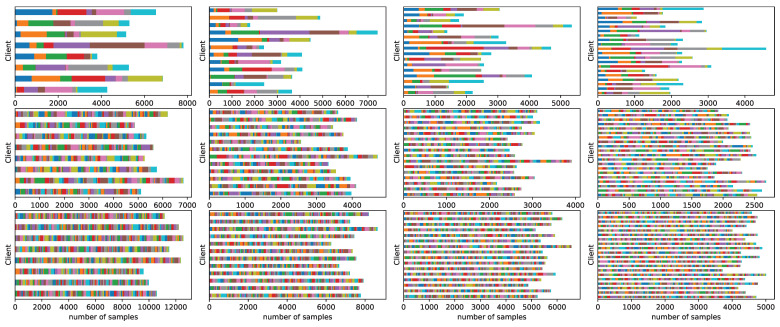
Simulated non-IID data over FL clients where different colors represent different classes. From top to bottom: CIFAR-10, CIFAR-100, and Tiny-ImageNet. From left to right: 8, 12, 16, and 20 clients.

We consider federated learning scenarios with different numbers of clients, specifically 8, 12, 16, and 20. To achieve the statistical heterogeneity, we partition CIFAR-10, CIFAR-100 and Tiny-ImageNet in a non-IID manner. All three datasets follow the same partitioning method. Specifically, prior to federated data partitioning, we randomly select 10% training samples from the original training dataset to serve as the public unlabeled dataset $D_{S}$ on the server, whose original labels are deliberately discarded. The remaining 90% of the original training dataset is then partitioned across clients following the latent Dirichlet allocation (LDA) partitioning with α=0.5 [45] to build non-IID federated datasets. The simulated non-IID data over FL clients is depicted in Figure 7. Furthermore, within each client, the allocated private dataset is further divided into private training dataset $D_{i}^{trn}$ and private validation dataset $D_{i}^{val}$, based on a split ratio of 8 to 1. At the same time, the original test dataset is similarly partitioned into clients following a consistent label distribution with the partitioned training dataset.

To reflect the resource heterogeneity, each client is allocated computational resources proportionate to the amount of their respective private data ${\left\{\left|D_{i}\right|\right\}}_{i=1:N}$, ensuring that the client with the least amount of private data can only support a few of the smallest candidate architectures in terms of FLOPs, whereas the client with the largest proportion of data is compatible with all candidate architectures within the search space A.

**Supernet training settings:**

The knowledge model θ is designated as the smallest subnet $\alpha_{s}$ within our search space *A*. The loss function L is set to class-balanced softmax function [46] to alleviate the impact of statistical heterogeneity. The divergence function $L_{div}$ is set to KL divergence with a temperature of 1.0 in ensemble distillation and inplace distillation when solving the optimization problem (6). The total number of federated training rounds is set to be 120. The federated supernet training process requires about 3.5 to 7.6 h, depending on the dataset complexity and the number of clients.

**Search settings:**

Our personalized architecture search is based on the evolution algorithm as shown in Algorithm 2. We set the population size $N_{pop}$ to 256, with 128 new individuals generated by mutation and 128 by crossover in each generation to 128 and 128. A total of 20 generations are specified for each run, so each search explores 5376 candidate architectures.

Additionally, a random forest consisting of 100 regression trees with a maximum depth of 15 is employed as the performance predictor to accelerate the search process for each FL client. The mean squared error (MSE) is used as the splitting criterion within the trees. As detailed in Algorithm 2, the candidate architectures and their corresponding validation accuracies from the initial population of the evolution algorithm are utilized to train the performance predictor for each client. Specifically, each candidate architecture is encoded as a vector by sequentially stacking the kernel size, expansion ratio, and the number of channels of each layer. That is, the architectural parameters of all layers are flattened and concatenated into a single vector representation. The corresponding test accuracy of each candidate architecture is obtained by inheriting the supernet weights, calibrating BN statistics on the client’s private training dataset, and then evaluating on the client’s private validation dataset. Collecting the architecture–accuracy pairs and then training a random-forest-based predictor is highly efficient, which takes only about 10 min. Consequently, the evaluation of candidate architectures in the remaining evolutionary rounds can be rapidly performed by the trained predictor, with negligible computational overload. Benefiting from the predictor, the complete search process for each client takes only about 20 min.

**Federated retraining settings:**

During the retraining stage, we optimize all models with a general SGD optimizer with a momentum of 0.9 and a weight decay of $4\times 10^{-5}$. The learning rate is 0.1 with a batch size of 256 and decays with cosine annealing. The total number of federated training rounds is set to be 60. The federated retraining process requires approximately 0.5 to 2.0 h, depending on the dataset complexity and the number of clients.

### 5.2. Effectiveness of Resource-Resilient Supernet Training

As stated in Section 4.1, the proposed resource-resilient federated supernet training strategy effectively enhances the stability of the supernet, thereby improving the accuracy of performance ranking among candidate architectures. To verify this claim, we conduct experiments on the CIFAR-100 dataset. Specifically, we partition the search space into 19 bins by the FLOPs of subnets and randomly sample one subnet from each bin. Then, all 19 architectures are trained from scratch on public dataset $D_{S}$ and evaluated on the original CIFAR-100 test dataset to obtain their ground truth performance ranking.

We train supernets separately using two federated supernet training strategies: (1) our proposed resource-resilient strategy, and (2) the resource-aware strategy adopted by existing works [11,12,13]. Additionally, we further consider a resource-agnostic federated supernet training strategy that ignores resource constraints, allowing free subnet assignment across all clients. Note that, in resource-agnostic strategy, all subnets can be viewed as updating based on the joint data distribution over clients. Then, we evaluate the ability of the trained supernets to serve as performance proxies in predicting performance rankings of the above 19 subnets. Moreover, we also include two strong baselines—FLOPs and the number of parameters (#params)—which are widely recognized as reliable indicators of performance ranking [47].

**Figure 8:**
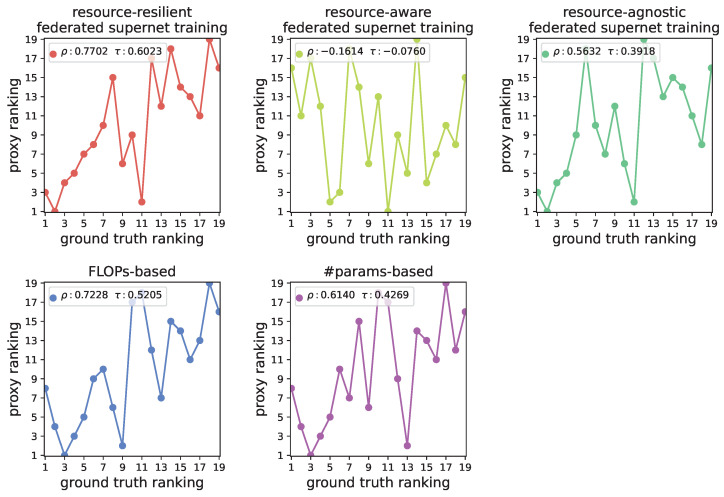
Ranking correlation between ground truth performance and proxy performance across 19 candidate architectures.

Figure 8 gives the correlation results between these proxy performance rankings and the ground truth performance ranking, where ρ and τ denote the Spearman and Kendall rank correlation score, respectively. Firstly, as illustrated in Figure 8, the supernet trained using the resource-aware strategy exhibits the worst performance ranking correlation, due to exacerbated gradient conflicts caused by the coupled resource and statistical heterogeneity. Secondly, compared to resource-aware strategy, the resource-agnostic strategy improves correlation because it ignores resource constraints. However, this resource-agnostic strategy still underperforms the two baseline proxies, FLOPs and #params. This is because the federated supernet training is still affected by statistical heterogeneity across clients. Finally, and most importantly, the supernet trained using our proposed resource-resilient strategy achieves the most precise performance ranking. It significantly outperforms the other federated supernet training strategies and surpasses the two baselines. These results clearly demonstrate the effectiveness of our proposed resource-resilient federated supernet training strategy in the heterogeneous FL scenarios, thereby enabling more reliable guidance for the personalized architecture search.

### 5.3. Effectiveness of Performance Predictor

**Figure 9:**
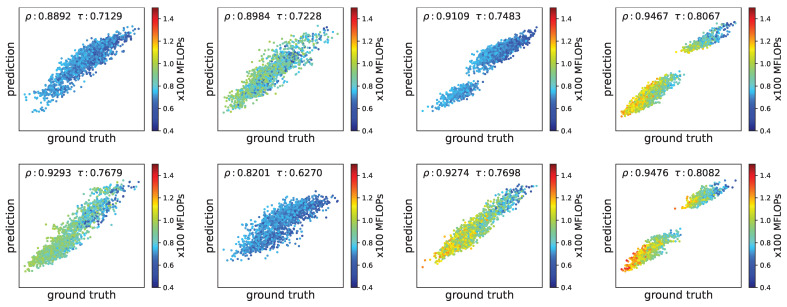
Ranking correlation between the ground truth performance and the predicted performance by random-forest-based performance predictors across eight clients. Each point represents a candidate architecture, with color indicating its FLOPs as shown in the colorbar.

As described in Section 4.2, we train a random-forest-based performance predictor to accelerate the architecture search on each client. To assess the predictor’s prediction capability, we randomly sampled 2000 architecture-performance pairs via reject sampling for each client, with 10% (i.e., 200 samples) samples used for training the predictor and the remaining 90% used for testing. The prediction results across eight heterogeneous clients are illustrated in Figure 9.

As shown in Figure 9, the predicted performance exhibits a high ranking correlation with the ground truth across all clients. This demonstrates that the random-forest-based predictor generalizes well even with limited training data. This confirms the effectiveness of the predictor in guiding the search process.

### 5.4. Effectiveness of heteroFedAvg

In this section, we evaluate the effectiveness of our model-heterogeneous FL algorithm, heteroFedAvg, based on the CIFAR-100 dataset. For comparison, we include two typical model-heterogeneous FL algorithms based on knowledge distillation, FedKEMF [35] and FedMD [26]. All methods are evaluated under both model-heterogeneous and model-homogeneous settings. Specifically, in the heterogeneous setting, client are assigned models randomly sampled from the search space A based on their individual resource constraints. In contrast, the homogeneous setting adopts the smallest subnet $\alpha_{s}\in A$—which is universally compatible with all clients—as the shared model across clients. All experiments follow the setup described in Section 5.1, with the number of clients fixed to 8. The average Top-1 accuracy across FL clients for each algorithm is reported in Table 1.

In the model-heterogeneous FL scenario, our heteroFedAvg performs competitively with FedKEMF and FedMD. Furthermore, in the model-homogeneous FL scenario, our proposed heteroFedAvg degrades to FedAvg and outperforms FedKEMF and FedMD by approximately 2% average Top-1 accuracy. This suggests that direct parameter aggregation achieves more effective information fusion than knowledge distillation. Overall, these results demonstrate that our proposed heteroFedAvg is both effective and generalizable.

### 5.5. Effectiveness of HAPFNAS

Next, we conduct extensive experiments across 3 datasets and 4 client numbers, totaling 12 FL tasks, to comprehensively evaluate the effectiveness of the proposed HAPFNAS method. Specifically, our HAPFNAS trains the supernet based on the proposed resource-resilient federated supernet training strategy, then performs predictor-guided personalized evolutionary NAS to search for the optimal personalized models for heterogeneous clients. To explore the direct impact of the supernet’s ranking capability on the performance of searched models, we introduce two other federated supernet training strategies: (1) ‘resource-aware’: the resource-aware strategy adopted by existing works [11,12,13], and (2) ‘resource-agnostic’: a resource-agnostic strategy which disregards resource constraints and enables unrestricted subnet assignment among clients. In addition, two strong non-NAS baselines are included: (1) ‘FLOPs-based’: randomly sampled subnets that best match the resource constraint of each client, equivalent to the results searching using FLOPs as the performance proxy; (2) ‘homogeneous’: a setting where all clients share the same smallest subnet $\alpha_{s}\in A$ that is compatible with every client. All retraining experiments are based on the heteroFedAvg algorithm for fairness. The average Top-1 accuracy of searched models across clients are reported in Table 2.

From Table 2, it is interesting to note that, in most tasks, ‘homogeneous’ obtains the worst average Top-1 accuracy. This implies that forcing all clients to adopt a shared model for compatibility with the most constrained client leads to suboptimal performance in heterogeneous FL scenarios. In comparison, ‘FLOPs-based’ significantly outperforms ‘homogeneous’ by configuring the largest acceptable personalized model architecture for each client. However, as it does not consider the statistical heterogeneity, its performance remain suboptimal. This reflects that focusing solely on model size, while ignoring the local data distribution, falls short in designing effective personalized model.

As shown in Table 2, when NAS is employed in the design of heterogeneous personalized model architectures, ‘resource-aware’ and ‘resource-agnostic’ exhibit similar performance across 12 tasks. In many tasks, both ‘resource-aware’ and ‘resource-agnostic’ can only search for decent architectures and do not produce significant performance advantages compared to ‘FLOPs-based’. Furthermore, it is obvious that our HAPFNAS outperforms the other strategies in most tasks. This indicates that our proposed resource-resilient federated supernet training strategy brings advantages to the searched results by improving the stability and accuracy of the supernet’s performance ranking, compared to ‘resource-aware’ and ‘resource-agnostic’ strategies.

It is also worth noting that, when the heterogeneous FL task is simple, such as tasks on the CIFAR-10 dataset, our HAPFNAS only obtains competitive performance with other methods. When the heterogeneous FL task is more complex, particularly in settings with more clients (e.g., 16 or 20) and more challenging datasets (e.g., CIFAR-100 and Tiny-ImageNet), our HAPFNAS shows more stable performance advantages in terms of the average Top-1 accuracy. This suggests that the more heterogeneous the FL task is, the more it requires personalized model architectures that fully match the resource characteristics and data distribution of FL clients.

**Statistical significance test**: To assess whether significant differences in average Top-1 accuracy exist among the compared strategies, we employ the Friedman test [48]. At a significance level α=0.05, the critical value equals 2.58, given 5 compared strategies across 12 tasks (3 datasets, 4 client numbers). The calculated Friedman statistic $F_{F}=11.89$. Since $F_{F}>2.58$, we reject the null hypothesis, indicating that there exist statistically significant differences among the evaluated strategies.

**Figure 10:**
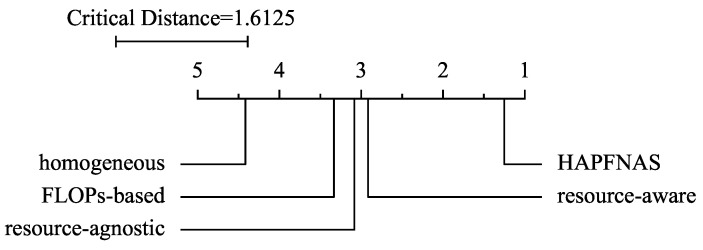
Bonferroni–Dunn test on different strategies, where average ranks of different strategies over 12 tasks (3 datasets, 4 client numbers) are presented.

To further investigate pariwise differences, we adopt the Bonferroni–Dunn test [48], treating our HAPFNAS as the control. At a significance level α=0.05, the critical distance is calculated to be 1.6125, given 5 compared strategies across 12 tasks (3 datasets, 4 client numbers). The corresponding critical difference (CD) diagram is shown in Figure 10. According to the results, our HAPFNAS achieves the highest average rank among the five evaluated strategies. Furthermore, it demonstrates statistically significant performance improvements compared to the ‘resource-aware’, ‘resource-agnostic’, ‘FLOPs-based’, and ‘homogeneous’.

## 6. Conclusions

In this work, we have proposed a heterogeneity-aware personalized federated neural architecture search method, for heterogeneous FL scenarios, termed HAPFNAS. By integrating knowledge distillation techniques, our framework enables stable one-shot supernet training in federated learning environments, while effectively utilizing distributed private data from participating FL clients. Moreover, an aggregation algorithm for heterogeneous models originating in the same search space has been developed, thereby facilitating model-heterogeneous federated learning. Extensive experimental results demonstrate that our HAPFNAS can successfully discover promising personalized model architectures for FL clients, thus verifying its effectiveness.

## Figures and Tables

**Table 1:** Average Top-1 accuracy comparison of heteroFedAvg with other model-heterogeneous federated learning algorithms.

Methods	Average Top-1 Accuracy Across 8 Clients (%)	
	Heterogeneous Models	Homogeneous Models
FedKEMF [35]	68.94	66.07
FedMD [26]	68.86	66.18
HeteroFedAvg (proposed)	**69.36**	**68.18**

**Table 2:** Average Top-1 accuracy comparison of HAPFNAS with other methods across 12 tasks (3 datasets, 4 client numbers). ^†^: Homogeneous models are deployed on heterogeneous FL clients.

Dataset	*N* Clients	Average Top-1 Accuracy Across *N* Clients (%)				
		HAPFNAS	Resource-Aware	Resource-Agnostic	FLOPs-Based	Homogeneous ^†^
CIFAR-10 [41]	N=8	88.33	88.29	**88.74**	88.18	85.35
	N=12	85.38	**86.01**	84.76	84.87	83.39
	N=16	84.41	**84.49**	83.00	83.39	83.27
	N=20	**79.28**	78.52	77.76	78.14	79.11
CIFAR-100 [41]	N=8	**69.36**	68.49	68.92	68.34	65.46
	N=12	**64.73**	63.12	64.13	62.93	61.96
	N=16	**58.81**	57.78	57.34	56.35	56.31
	N=20	**55.25**	53.63	52.87	54.39	53.20
Tiny-ImageNet [42]	N=8	**48.64**	47.31	47.55	47.75	46.26
	N=12	**46.47**	45.96	46.24	45.68	44.37
	N=16	**45.45**	43.56	43.90	44.55	43.45
	N=20	**42.10**	40.78	41.34	40.88	41.19

## Data Availability

Data and code are available at a GitHub repository: https://github.com/variant-star/FederatedScopeNAS, accessed on 13 July 2025.
